# Understanding COVID-19 Vaccine Uptake and Hesitancy among People with HIV in Freetown, Sierra Leone: A Cross-Sectional Study

**DOI:** 10.3390/vaccines11111685

**Published:** 2023-11-02

**Authors:** Peterlyn E. Cummings, Sulaiman Lakoh, Sahr A. Yendewa, Samuel P. E. Massaquoi, Peter B. James, Foday Sahr, Gibrilla F. Deen, Robert A. Salata, Pelema Gevao, George A. Yendewa

**Affiliations:** 1 College of Medicine and Allied Health Sciences, University of Sierra Leone, Freetown 00232, Sierra Leone; eyamide00232@gmail.com (P.E.C.); lakoh2009@gmail.com (S.L.); fsahr65@gmail.com (F.S.); gibrilladeen1960@yahoo.com (G.F.D.); ppgevao34@gmail.com (P.G.); 2 Connaught Hospital, University of Sierra Leone Teaching Hospitals Complex, Ministry of Health and Sanitation, Freetown 00232, Sierra Leone; 3 Ministry of Health and Sanitation, Freetown 00232, Sierra Leone; syendewa@gmail.com (S.A.Y.); drspem@gmail.com (S.P.E.M.); 4 Faculty of Health, Southern Cross University, Lismore, NSW 2480, Australia; peter.james@scu.edu.au; 5 Department of Medicine, Case Western Reserve University School of Medicine, Cleveland, OH 44106, USA; robert.salata@uhhospitals.org; 6 Division of Infectious Diseases and HIV Medicine, University Hospitals Cleveland Medical Center, Cleveland, OH 44106, USA; 7 Republic of Sierra Leone Armed Forces, Department of Defense, Freetown 00232, Sierra Leone; 8 Johns Hopkins Bloomberg School of Public Health, Baltimore, MD 21205, USA

**Keywords:** COVID-19, vaccination, HIV, Sierra Leone

## Abstract

People with HIV (PWH) incur a higher risk of COVID-19-related morbidity and mortality rates, yet less is known about COVID-19 vaccine uptake and hesitancy in this group. We conducted a cross-sectional study in Freetown, Sierra Leone, from April to June 2022, using the VAX scale, a validated instrument, to assess attitudes towards COVID-19 vaccination and calculate the hesitancy (VAX) scores. We used generalized linear models to identify the factors associated with vaccine hesitancy. Overall, 490 PWH were enrolled (71.4% female, median age: 38 years, median CD4 count: 412 cells/mm^3^). About 17.3% received ≥1 dose of a COVID-19 vaccine. The mean VAX score was 43.14 ± 7.05, corresponding to 59.9% participants being vaccine-hesitant. A preference for natural immunity (65.8%) and concerns about profiteering (64.4%) were the commonest reasons for hesitancy, followed by a mistrust of vaccine benefits (61.4%) and worries about future effects (48.0%). In the adjusted regression analysis, being a Muslim (β = 2.563, *p* < 0.001) and having an urban residence (β = 1.709, *p* = 0.010) were associated with greater vaccine hesitancy, while testing for COVID-19 was associated with reduced vaccine hesitancy (β = −3.417, *p* = 0.027). These findings underscore the importance of addressing vaccine hesitancy as a critical element boosting COVID-19 vaccine uptake among PWH.

## 1. Introduction

More than three years since the first cases were reported, the coronavirus disease 2019 (COVID-19) pandemic, caused by severe acute respiratory syndrome coronavirus 2 (SARS-CoV-2), continues to pose a significant global challenge, with millions of confirmed cases and documented deaths reported worldwide [1]. Unlike previous health crises of a similar scale, the development of vaccines occurred relatively early in the pandemic, leading to a significant shift in its trajectory. The current COVID-19 vaccines have proven to be effective in reducing virus transmission [2,3], severity of illness [4], and COVID-19-associated mortality [5]. COVID-19 vaccination is recommended at present for most individuals, including people living with HIV (PWH), who are a priority population for vaccination. Compared with their non-HIV counterparts, PWH may have an increased susceptibility to severe illness and poor outcomes from SARS-CoV-2 infection, partly due to having a higher prevalence of premorbid risk factors, including cardiovascular diseases, diabetes, and obesity [6,7].

Despite the ongoing efforts to promote COVID-19 vaccination, hesitancy to vaccinate persists among various populations, including PWH. Studies have provided insights into COVID-19 vaccination status and hesitancy among PWH. In a large global HIV cohort enrolled in the REPRIEVE study (*n* = 6952), Fulda et al. [8] reported a COVID-19 vaccination rate of 55%, with considerable disparities in the vaccination coverage rates noted between high-income countries in North America and Europe (71%) and low-income countries in Sub-Saharan Africa (18%). In a separate study assessing the reasons for COVID-19 vaccine hesitancy among PWH (*n* = 1030), Shrestha et al. [9] found that up to 90% of the survey respondents in the United States experienced some degree of COVID-19 vaccine hesitancy, with a greater reluctance to vaccinate associated with being Black, holding conservative political viewpoints, and having concerns about vaccine safety and efficacy. However, the determinants of COVID-19 vaccine hesitancy among PWH in low-income settings are understudied, especially in Sub-Saharan African countries where the global burden of HIV is highest.

With the emergence of more infectious variants of SARS-CoV-2, PWH in regions with a high burden of HIV may face continued risks of COVID-19-related morbidity and mortality, which may necessitate additional booster doses of the COVID-19 vaccination. Understanding and addressing the factors contributing to vaccine hesitancy among PWH is essential to enhance the vaccination rates in this population. In this study, we aimed to assess COVID-19 vaccination coverage and hesitancy in a cohort of PWH in Freetown, Sierra Leone.

## 2. Methods

### 2.1. Study Design, Population, and Setting

We conducted a cross-sectional study to assess COVID-19 vaccination coverage and hesitancy among PWH who received routine clinical care at the HIV Clinic at Connaught Hospital in Freetown, Sierra Leone, from April to June 2022. The HIV Clinic is the largest HIV treatment center in Sierra Leone and has over 4000 PWH in active clinical follow-ups. Connaught Hospital is affiliated with the College of Medicine and Allied Health Sciences of the University of Sierra Leone. The study inclusion criteria were (1) age ≥ 18 years, (2) documented evidence of HIV infection, and (3) willingness to provide informed consent. The exclusion criteria were (1) age < 18 years and (2) unwillingness or inability to provide informed consent. Eligible patients were approached during routine clinic visits and informed of the purpose of the study. We used convenience sampling to enroll interested participants.

### 2.2. Sample Size Calculation and Justification

We estimated the minimum sample size n, according to Lwanga and Lemeshow [10], as follows:

n = Z^2^ × p (1 − p)/e^2^

where Z = 1.96 at a 95% confidence interval (CI), p = prevalence of COVID-19 vaccine hesitancy among PWH in Sierra Leone, and e is the error rate. Given the lack of studies on PWH from Sierra Leone, we used a COVID-19 vaccine hesitancy rate of 50% and an error rate of 5%, which yielded a sample size of 384. Factoring in a 10% non-response rate yielded a final minimum sample size of 422, which was sufficiently powered at 80% to detect associations between the variables using a 2-tailed test.

### 2.3. Survey Instrument, Procedures, and Measures

We collected baseline sociodemographic, clinical, and health data. The sociodemographic variables were collected from participants using a self-reporting method and included age, sex, highest education attained, occupation, and religion. Clinical data were collected from the patient’s medical records. HIV-specific data included the most recent CD4 count (dichotomized as <200 cells/mm3 vs. ≥200 cells/mm3), HIV viral load (dichotomized as <1000 copies/mL vs. ≥1000 copies/mL), antiretroviral therapy (ART), and duration since HIV diagnosis. We collected and verified the data for COVID-19 vaccination status from vaccination records or the patient’s charts. For COVID-19-related experiences, we collected the data on SARS-CoV-2 testing history and vaccine-related adverse effects based on self-reports.

The instrument for assessing COVID-19 vaccine hesitancy was adapted from the 12-item Vaccination Attitudes Examination (VAX) scale originally developed by Martin and Petrie [11], which we previously validated in Sierra Leone to assess COVID-19 vaccine hesitancy among healthcare workers [12]. Briefly, the VAX scale assesses attitudes towards vaccines across four domains, as follows: (1) a mistrust of vaccine benefits, (2) worries about unforeseen future effects, (3) concerns about commercial profiteering, and (4) preference for natural immunity [11]. Items are rated on a six-point Likert scale with equidistant scores, as follows: 1 = strongly agree, 2 = agree, 3 = slightly agree, 4 = slightly disagree, 5 = disagree, and 6 = strongly disagree. The instrument was first piloted to the target study population (*n* = 10) to ensure the clarity of items. The pilot survey participants were not included in the final study.

We estimated the prevalence of COVID-19 vaccine hesitancy by summing the participant responses to each item on the VAX scale. As items 4–12 were negatively worded, we reverse-scored the responses to ensure that all items were keyed in a positive direction, with higher VAX scores indicating a greater reluctance to vaccinate against COVID-19. The possible VAX scores ranged from 12 (positive attitude) to 72 (negative attitude). As previously described by us and others [12,13], VAX scores ranging from 12–32 (i.e., 25th percentile) were categorized as low hesitancy, scores ranging from 33–52 (i.e., 50th percentile) were classified as moderate hesitancy, while scores >52 (i.e., 75th percentiles) indicated high COVID-19 hesitancy. Vaccine hesitancy was defined as VAX score > mean (i.e., 50th percentile). The normality of the VAX score distribution was assessed by examining the histogram, and normality was considered to be achieved if the absolute skewness value was ≤2 or if the absolute kurtosis (excess) was ≤4, which assumed the homogeneity of variances under the null hypothesis [14].

To assess the psychometric properties of the survey instrument, we estimated the internal consistency of the responses using mean inter-item reliability correlations and Cronbach’s alpha coefficients (α), with an overall α > 0.7 regarded as acceptable. We performed an exploratory factor analysis using the principal component analysis with an orthogonal (Varimax) rotation to assess the dimensional structure of the VAX scale.

### 2.4. Statistical Analysis

Statistical analyses were performed using the SPSS Version 29.0 (Armonk, NY, USA; IBM Corp). Categorical variables were reported as frequencies (percentages) and continuous variables as means (standard deviation) or medians (range or interquartile range, IQR). Generalized linear regression models were used to identify the factors associated with COVID-19 vaccine hesitancy, represented by VAX scores. The covariates tested included sociodemographic and clinical data, as previously described, and were included in the multivariable model if significant in the univariate model. In all the analyses, the statistical significance was set at *p* < 0.05.

### 2.5. Ethical Approval

Ethical approval was obtained from the Sierra Leone Ethics and Scientific Review Committee (approval date: 20 December 2021). Written informed consent was obtained from each participant before enrolment in the study.

## 3. Results

### 3.1. Characteristics of Participants

A total of 490 PWH participated in the study (Table 1), of which 71.4% (350/490) were female. The median age was 38 years (IQR 32–49) and the majority were single (60.6%, 297/490), employed in the informal sector (69.4%, 340/490), and Muslim (69.2%, 339/490). Most (80%, 392/490) had attained a primary education or higher. The median CD4 count was 412 cells/mm^3^ (IQR 256–508). Most (83.9%, 411/490) were virologically suppressed (<1000 copies/mL) and on dolutegravir-based ART (57.8%, 283/490).

### 3.2. COVID-19 Vaccine Uptake and Experiences

As shown in Table 1, 17.3% (85/490) of the participants received a COVID-19 vaccine. Of these, 63.5% (54/85) received one dose of the COVID-19 vaccine, while 36.5% (31/85) received a complete series of vaccinations (i.e., two doses). Furthermore, 38.8% (33/85) received the Janssen (Johnson & Johnson) vaccine, 32.9% (28/85) received the AstraZeneca vaccine, and 28.2% (24/85) received the Sinopharm vaccine. About 44.6% (37/85) reported experiencing at least one vaccine-related adverse effect (not mutually exclusive): pain at the injection site (38.8%, 33/85), body aches (9.4%, 8/85), and fever (3.5%, 3/85). Overall, only 5.7% (28/490) had been tested for SARS-CoV-2 infection, of which no positive cases were detected.

### 3.3. Prevalence of COVID-19 Vaccine Hesitancy

**Figure 1:**
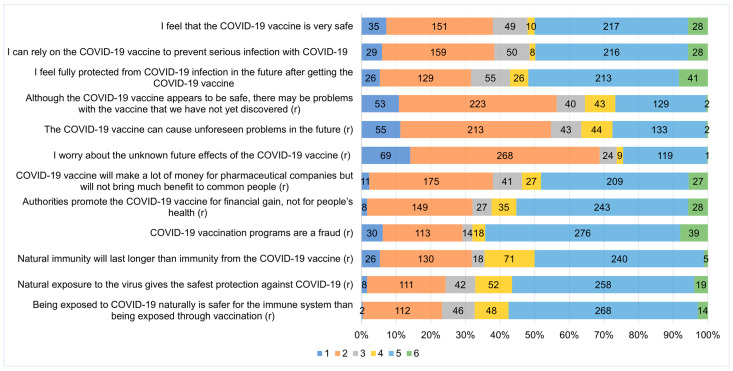
Responses to COVID-19 vaccine hesitancy questions among PWH. Abbreviations: (r), reverse-scored in a positive direction on a 6-point Likert scale ranging from 1 (strongly disagree) to 6 (strongly agree) and reclassified hesitancy as low (1 and 2), moderate (3 and 4), and high (5 and 6).

Figure 1 shows the distribution of individual responses to the participants’ responses to items on the VAX scale, which demonstrated excellent internal consistency (Cronbach’s α = 0.94 overall, range: 0.88–0.94 across domains) (Table 2). Similarly, the mean inter-item correlation was high (r = 0.645 overall, range: 0.745–0.892 across domains). Exploratory factor and principal component analyses using Varimax rotation confirmed the four-factor solution of the original VAX scale by Martin and Petrie [11].

The overall mean VAX score was 43.14 ± 7.05, which coincided with the median of 44 (minimum 27, maximum 60) and met the histogram test for normality. Additionally, the absolute value of the skewness was 0.35 and the absolute value of the kurtosis (excess) was 1.19, which agreed with the criteria for normality.

Based on the mean VAX score of 43.14, about 59.9% of participants were estimated as expressing COVID-19 hesitancy (Table 3). Across domains, a preference for natural immunity (65.8%, mean score: 11.84 ± 3.89) and concerns about commercial profiteering (64.4%, mean score: 11.62 ± 4.11) were the most common reasons for vaccine hesitancy, followed by a mistrust of vaccine benefits (61.4%, mean score: 11.06 ± 4.59) and worries about unforeseen future effects (48.0%, mean score: 8.62 ± 4.05). Furthermore, 6.7%, 91.0%, and 2.3% of the participants were classified into the low-, mild-to-moderate-, and high-level vaccine hesitancy categories, respectively.

### 3.4. Factors Associated with COVID-19 Vaccine Hesitancy

In the univariate and multivariable linear regression analyses (Table 4), greater COVID-19 vaccine hesitancy was associated with being Muslim (β = 2.563, *p* < 0.001) and residence in urban areas (β = 1.709, *p* = 0.010), while being tested for COVID-19 was associated with reduced vaccine hesitancy (β = −3.417, *p* = 0.027).

## 4. Discussion

Despite being vulnerable to poor outcomes from COVID-19, there are limited studies on COVID-19 vaccine uptake and hesitancy among PWH in Sub-Saharan Africa. To effectively address this issue in the region, it is crucial to measure and understand the extent and underlying reasons for this phenomenon. Our study revealed a concerningly low COVID-19 vaccine coverage (17.3%) and a high level of vaccine hesitancy (59.9%) among PWH in an urban setting in Sierra Leone. Similar findings were reported in a study from South Africa (*n* = 213) by Govere-Hwenje et al. [15], where 57% of PWH indicated a willingness to receive a COVID-19 vaccination, while 21% were unwilling to vaccinate, and 20% were unsure. Another study (*n* = 660) by Sulaiman et al. [16] observed a 58% COVID-19 vaccine hesitancy rate among PWH across six hospital systems in Nigeria. In contrast, Muhindo et al. [17] found a high COVID-19 vaccination coverage (69.6%) and high confidence in the vaccine in a Ugandan HIV cohort. Overall, however, these studies highlight the persistently high levels of COVID-19 vaccine hesitancy among PWH, despite concerted efforts to increase the vaccine uptake in this population.

The most common reasons for vaccine hesitancy among our study participants were a preference for natural immunity (65.4%) and concerns about the commercial profiteering/mistrust of pharmaceutical industries (64.4%). Notably, fewer participants expressed concerns about potential vaccine side effects (48.0%), in contrast with our previous study among healthcare workers in Sierra Leone, which was conducted during the same timeframe as the present study (*n* = 592), where 76% expressed worries about vaccine side effects [12]. We hypothesized that a HIV-positive status may introduce additional concerns, including uncertainty about vaccine safety and efficacy, and the possibility of vaccine interactions with HIV medications, which may affect the immune system [18,19]. Additionally, PWH may have negative experiences, including stigmatization, while navigating the healthcare system, which can contribute to their mistrust of the system and an erosion of confidence in healthcare authorities [20,21]. The combined influence of these intersectional factors, along with misinformation and conspiracy theories, may contribute to vaccine hesitancy among PWH. Interestingly, however, contrary to other studies [9,22], vaccine hesitancy was not predicted by HIV-specific factors, such as CD4 count or viral load suppression.

Urban residence was associated with higher COVID-19 vaccine hesitancy. However, a comprehensive study of the general population in five West African countries (Burkina Faso, Guinea, Mali, Senegal, and Sierra Leone) with a large sample size (*n* = 4198) revealed no significant association between vaccine acceptance and urban versus rural residence [23]. These contrasting findings may be attributed to various social, cultural, and economic factors that influence vaccine attitudes and behaviors in different settings. Urban areas, with their diverse populations comprising individuals with varying levels of education, income, and healthcare access, may have more complex vaccine decision-making processes [23]. Additionally, urban areas may be more susceptible to misinformation and conspiracy theories about vaccine safety and efficacy, contributing to vaccine hesitancy [24]. Further research is needed to explore the complex relationships between area of residence, vaccine hesitancy, and other socio-demographic factors.

Furthermore, it was noteworthy that being Muslim was a predictor of COVID-19 vaccine hesitancy in our study. This observation aligns with prior reports both from and outside Sub-Saharan Africa, which document increased levels of vaccine hesitancy among Muslim communities in the context of COVID-19 and other infectious diseases [18,25,26,27]. For instance, we observed in a recent study that children of Muslim parents had lower odds of completing the hepatitis B vaccination series for infants in Sierra Leone and Guinea, two countries with majority Muslim populations [27]. Similarly, a recent systematic review and meta-analysis conducted in the United Kingdom noted that being a Muslim parent was associated with decision making regarding routine childhood vaccinations [28]. Studies attempting to explain vaccine acceptance in Muslim communities have suggested apprehensions concerning the halal (permissible) nature of vaccines as a prominent factor in a willingness to receive a vaccine [29]. Others highlighted the influence of religious health fatalism in some Muslim communities, which posits that health outcomes are predetermined by God or a higher power, as playing a role in vaccine acceptance [30,31,32].

Nevertheless, it must be emphasized that the findings of these studies require nuanced interpretations, as there is no definitive evidence indicating that Muslims are universally less inclined to vaccinate when compared to individuals from different religious or cultural backgrounds. For example, a study from Malaysia, a Muslim-majority country, revealed that the majority of Muslim parents surveyed expressed positive attitudes towards childhood immunizations and believed that vaccines were permissible under Islamic law [29]. Conversely, COVID-19 vaccine hesitancy and a hesitancy towards other vaccines has been found among Christians, Hindus, and people of all religious backgrounds, suggesting that the relationship between religious identity, religiosity, and vaccine hesitancy is not universal and may be influenced by cultural, socioeconomic, and geographic contexts [28,33,34,35]. In these other groups, religious health fatalism has also been described as a major contributor to vaccine hesitancy and attitudes towards health in general [36,37]. Thus, efforts to address vaccine hesitancy among populations should focus on providing accurate information, addressing misconceptions, and building trust in the safety and efficacy of vaccines within these communities. Collaborative initiatives with trusted religious leaders, community organizations, and healthcare providers who understand the cultural and religious nuances can be effective in addressing vaccine hesitancy among populations with diverse religious and cultural backgrounds [25,37].

Another important finding was that being tested for COVID-19 was associated with lower vaccine hesitancy. This may be explained by theories of health behavior, which posit that positive health behavior may be elicited by first-hand experiences of illnesses [38,39,40]. For example, a personal experience of testing positive for COVID-19 or witnessing loved ones’ illnesses may enhance an individual’s perception of the severity of the infection, resulting in a heightened sense of urgency to protect oneself and others through vaccination [41]. Additionally, testing efforts often provide accurate information about the virus and vaccines, which can address misinformation and misconceptions that contribute to vaccine hesitancy [42]. Moreover, the emphasis on collective responsibility and community health in testing efforts may foster a sense of social obligation to take preventive measures, including vaccination [42,43].

Our study was characterized by strengths and limitations. Firstly, our survey utilized convenience sampling, which may have led to an underestimation of the true prevalence of COVID-19 vaccine hesitancy. Secondly, our study was limited to PWH in an urban setting and may not be representative of attitudes towards COVID-19 vaccination nationally. Thirdly, we collected the data on COVID-19 experiences from self-reporting cases. We acknowledge that this may have introduced recall bias. Lastly, our investigation of the barriers to vaccine uptake was not comprehensive and could be better explored using qualitative or mixed-methods study designs, which are better suited to explore causal links. Nevertheless, our study adds to our understanding of COVID-19 vaccination uptake and hesitancy among PWH in Sierra Leone and can help guide interventions aimed at improving COVID-19 vaccine acceptance in this population.

## 5. Conclusions

In summary, we observed a low high prevalence of COVID-19 vaccination and high levels of vaccine hesitancy among PWH in Freetown, Sierra Leone. The most frequent reasons for COVID-19 vaccine hesitancy were a preference for natural immunity and concerns about commercial profiteering, followed by a mistrust of vaccine benefits and worries about future side effects. Being Muslim and living in an urban residence were associated with greater vaccine hesitancy, while being tested for COVID-19 was associated with reduced vaccine hesitancy. Given that PWH remain vulnerable to poor COVID-19 outcomes, our findings may help inform strategies aimed at increasing COVID-19 vaccine uptake in this setting.

## Figures and Tables

**Table 1:** Sociodemographic and health characteristics of participants (N = 490).

Characteristics	N (%)
Gender	
Male	140 (28.6)
Female	350 (71.4)
Age, years	
Median (IQR)	38 (32–49)
<25	16 (3.3)
25–34	144 (29.4)
35–44	167 (34.1)
45–54	99 (20.2)
≥55	64 (13.1)
Relationship status	
Single	297 (60.6)
Married	155 (31.6)
Widowed/separated	38 (7.8)
Highest education level attained	
None	98 (20.0)
Primary	111 (22.7)
Secondary	225 (45.9)
Tertiary	56 (11.4)
Occupation	
Unemployed	109 (22.2)
Informal	340 (69.4)
Formal	41 (8.4)
Religion	
Christian	151 (30.8)
Muslim	339 (69.2)
Time living with HIV, years	
Median (IQR)	5 (3–11)
≤2	69 (14.1)
3–5	180 (36.7)
6–10	109 (22.2)
≥11	132 (26.9)
Current CD4 count, cells/mm^3^	
Median	412 (256–508)
<200	79 (16.1)
≥200	405 (82.7)
Viral load, copies/mL	
<1000	411 (83.9)
≥1000	79 (16.1)
Current ART	
Dolutegravir-based	283 (57.8)
Efavirenz-based	146 (29.8)
Lopinavir-based	61 (12.4)
History of chronic illness	
Yes	24 (4.9)
No	466 (95.1)
Tested for COVID-19	
Yes	28 (5.7)
No	462 (94.3)
Received any COVID-19 vaccine	
Yes	85 (17.3)
No	405 (82.7)
COVID-19 vaccine doses received (*n* = 85)	
1 (partial dose)	54 (63.5)
2 (full dose)	31 (36.5)
COVID-19 vaccine types (*n* = 85)	
Janssen (Johnson & Johnson)	33 (38.8)
AstraZeneca	28 (32.9)
Sinopharm	24 (28.2)
Experienced adverse COVID-19 vaccine effects (*n* = 85)	
Yes	37 (44.6)
No	46 (55.4)
Type of vaccine adverse effects (not mutually exclusive) (*n* = 85)	
Pain at injection site	33 (38.8)
Bodyaches	8 (9.4)
Fever	3 (3.5)

**Table 2:** Summary of COVID-19 attitude statements and vaccine hesitancy levels (in percentages, %).

COVID-19 Vaccine Attitude Statements	Vaccine Hesitancy Level			Mean Inter-Item Correlation	Domain Cronbach’s Alpha
	Low	Moderate	High		
Mistrust of vaccine benefits					
I feel that the COVID-19 vaccine is very safe	37.9	12.0	50.1		
I can rely on the COVID-19 vaccine to prevent serious infection of COVID-19	38.1	11.8	49.8	0.892	0.94
I feel fully protected from the COVID-19 infection in the future after receiving the COVID-19 vaccine	31.6	16.7	51.7		
Worries about unforeseen future effects					
Although the COVID-19 vaccine appears to be safe, there may be problems with the vaccine that we have not yet discovered (r)	56.3	17.0	26.7		
The COVID-19 vaccine can cause unforeseen problems in the future (r)	54.7	17.8	27.1	0.823	0.90
I worry about the unknown future effects of the COVID-19 vaccine (r)	68.8	6.7	24.5		
Concerns about commercial profiteering					
The COVID-19 vaccine will make a lot of money for pharmaceutical companies but will not bring much benefit to common people (r)	37.7	13.9	48.4		
Authorities promote the COVID-19 vaccine for financial gain, not for people’s health (r)	32.0	12.6	55.4	0.745	0.88
COVID-19 vaccination programs are a fraud (r)	29.4	6.8	63.8		
Preference for natural immunity					
Natural immunity will last longer than immunity from the COVID-19 vaccine (r)	31.8	18.2	50.0		
Natural exposure to the virus provides the safest protection against COVID-19 (r)	24.3	29.8	45.9	0.846	0.93
Being exposed to COVID-19 naturally is safer for the immune system than being exposed through a vaccination (r)	23.3	19.2	57.5		

Abbreviations: (r), reverse-scored in a positive direction on a 6-point Likert scale ranging from 1 (strongly disagree) to 6 (strongly agree) and reclassified hesitancy as low (1 and 2), moderate (3 and 4), and high (5 and 6).

**Table 3:** Domain and overall vaccine hesitancy scores.

Variables	Expected Range of VAX Score	Median (Min–Max)	VAX Score or N	% Participants With VAX Score
Overall hesitancy				
Mean (SD)	12–72	44 (27–60)	43.14 ± 7.05	59.9
Hesitancy domains				
Mistrust of vaccine benefits	3–18	12 (3–18)	11.06 ± 4.59	61.4
Worries about unforeseen future effects	3–18	6 (3–18)	8.62 ± 4.05	48.0
Concerns about commercial profiteering	3–18	13 (3–18)	11.62 ± 4.11	64.4
Preference for natural immunity	3–18	13 (3–18)	11.84 ± 3.89	65.8
Categories of hesitancy				
Low	12–32	33	33	6.7
Moderate	33–52	446	446	91.0
High	>52	11	11	2.3

Abbreviations: N, sample size; Min, minimum; Max, maximum; SD, standard deviation; VAX score, vaccine hesitancy score.

**Table 4:** Univariate and multivariable linear regression correlates of COVID-19 vaccine hesitancy.

Variables	Univariate			Multivariable		
	β	S.E.	*p*-Value	β	S.E.	*p*-Value
Sociodemographic information						
Sex: male	−0.896	0.702	0.202			
Age (years)	0.040	0.030	0.179			
Relationship status: single	−0.010	0.648	0.988			
Education: none	0.661	0.791	0.403			
Religion: Muslim	2.511	0.676	<0.001	2.563	0.674	<0.001
Unemployed	0.552	0.761	0.469			
Residence: urban	1.476	0.671	0.028	1.709	0.661	0.010
HIV-related factors						
Time living with HIV	0.119	0.074	0.107			
CD4 count	0.001	0.001	0.293			
Viral load: suppressed	0.474	0.861	0.582			
ART class: dolutegravir-based	1.179	0.640	0.065			
COVID-19 and other health information						
Tested for COVID-19	−3.417	1.355	0.012	−2.943	1.334	0.027
History of chronic illness	0.356	1.467	0.808			

β, estimate; S.E., standard error.

## Data Availability

The data presented in this study are available on request from the corresponding author upon reasonable request.
